# Waist circumference percentiles among Turkish children under the age of 6 years

**DOI:** 10.1007/s00431-012-1822-5

**Published:** 2012-09-27

**Authors:** Nihal Hatipoglu, M. Mumtaz Mazicioglu, Serpil Poyrazoglu, Arda Borlu, Duygu Horoz, Selim Kurtoglu

**Affiliations:** 1 Department of Pediatric Endocrinology, Medical Faculty, Erciyes University, 38039 Kayseri, Turkey; 2 Department of Family Medicine, Medical Faculty, Erciyes University, Kayseri, Turkey; 3 Department of Public Health, Medical Faculty, Erciyes University Faculty of Medicine, Kayseri, Turkey

**Keywords:** Waist circumference reference values, Preschool children

## Abstract

Waist circumference, a proxy measure of abdominal obesity, is associated with cardio-metabolic risk factors in childhood and adolescence. Although there are numerous studies about waist circumference percentiles in children, only a few studies cover preschool children. The aim of this study was to develop age- and gender-specific waist circumference smoothed reference curves in Turkish preschool children to determine abdominal obesity prevalence and to compare them with reference curves obtained from different countries. The design of the study was cross-sectional. A total of 2,947 children (1,471 boys and 1,476 girls) aged 0–6 years were included in the study. The subjects were divided according to their gender. Waist circumference was measured by using a standardized procedure. The age- and gender-specific waist circumference reference curves were constructed and smoothed with LMS method. The reference values of waist circumference, including 3rd, 10th 25th, 50th, 75th, 90th, and 97th percentiles, and standard deviations were given for preschool children. Waist circumference values increased with age, and there were differences between genders. The prevalence of abdominal obesity was calculated as 10.1 % for boys and 10.7 % for girls. Having compared our data with two other countries’ data, we found that our waist circumference data were significantly lower. This is the first cross-sectional study for age- and gender-specific references of 0- to 6-year-old Turkish children. The gender- and age-specific waist circumference percentiles can be used to determine the risk of central obesity.

## Introduction

The prevalence of childhood overweight and obesity gradually increases, even in preschool-age children [29]. Globally, more than 20 million children who are below the age of 5 are overweight [44]. The finding that overweight in preschool children is an indicator of five times more overweight in adolescence and four times more overweight in adulthood is compared with normal-weight counterparts [28]. Since chronic conditions such as diabetes, cardiovascular disorders, hypertension, stroke, asthma, and certain neoplastic disorders may occur in late adulthood, prevention and early interventions for overweight or obesity are a real concern [5].

Although the body mass index (BMI) is used as a marker of obesity, it may be a less sensitive indicator in early childhood [32]. A few studies on preschool children showed a weak correlation between BMI and metabolic risk factors [19, 36]. Thus, it may be considered that BMI is an unreliable marker in this age group since central adiposity which is associated with metabolic risk factors is not adequately reflected by BMI. Therefore, early identification of children’s central adiposity is important [40, 41].

The waist circumference (WC), one of the anthropometric techniques, is strongly correlated with metabolic risk factors and now becomes widely used [23]. In addition, WC is an essential diagnostic criterion for metabolic syndrome according to the National Cholesterol Education Program (NCEP) Adult Treatment Panel III (ATP III) and the International Diabetes Federation (IDF) [40, 41]. There are recent reports indicating that the onset of risk factors for metabolic syndrome under the age of 10 years can be considered. The NCEP and IDF criteria for metabolic syndrome may be modified to explain metabolic risks under the age of 10 years [14, 30, 45]. Thus, WC cutoffs and reference values in preschool children can be useful for identifying children with metabolic risk and might be helpful for early intervention.

The preschool age is a difficult group for achievement and anthropometric measure while school-age children are easily accessible. Therefore, although many countries have their own reference values for WC [1, 17–21, 27, 35, 39], a few studies cover preschool-age children [11, 12, 21, 37].

The aim of this study was to establish reference values of waist circumference in Turkish preschool children. This study provided the prevalence of abdominal obesity in preschool children of both sexes. Furthermore, 50th and 90th percentiles of WC were compared with the ones in a few other countries [11, 37].

## Materials and methods

### Subjects

We analyzed the data of Anthropometry of the Turkish Children aged 0–6 years (ATCA-06). The ATCA-06 study was conducted from September 2009 to May 2010 in one of the five great cities of Turkey with about 1,200,000 residents. The sampling design of the study was a two-stage probability sampling for preschool children living in Kayseri.

The primary sampling unit was the family health centers (Aile Sagligi Merkezi, ASM) located in the city center and suburbs. At the first stage, children were selected from 21 ASMs in Kayseri by stratifying according to the socio-economic levels of their parents. Children aged 0–6 years old were randomly selected among the list of district midwives and they were invited to ASMs with their parents. Those infants and children, whose parents did not accepted, were invited again to participate in the study by midwives.

A total of 2,947 children (1,471 boys and 1,476 girls) whose parents gave consent were included in the study. After data collection, we removed 230 (119 boys and 110 girls, 7.9 % of sample size) subjects from the study because of reasons such as missing data in the interviewing forms, children with growth disorders, or using any kind of medication which could interfere with growth. Then, to obtain normally distributed data, the higher and lower limits (3rd–97th percentiles) for each gender and quarter age were removed (*n* = 115). Chronological age was calculated by subtracting the date of birth from the date of observation. Each quarter year elapsed from their birthday was noted. The WHO group recommended that early growth patterns should be documented in intervals shorter than 3 months [10, 13]. So, we calculated by age and gender in 0–6-year-old children in quarter-year intervals except for the 0–28-day newborn period.

The study protocol was approved by the Ethics Committee of Erciyes University. Parents’ written consent was obtained prior to the study and the procedures were in accordance with those outlined by the Declaration of Helsinki.

### Measurements

All measurements were done by well-trained technicians. Waist circumference was measured with a non-stretchable tape at the midpoint of the lowest rib cage and the iliac crest, to the nearest 0.1 cm, at the end of a gentle expiration [43]. The circumference was measured without clothing. Central obesity was defined as WC higher than the 90th percentile [24].

### Statistical analysis

Construction of the centile curves was performed with the LMS Chart Maker Pro version 2.3 software program (The Institute of Child Health, London), which fits smooth centile curves to reference data [8]. This method summarizes percentiles at each age based on the power of age-specific Box–Cox power transformations that are used to normalize data. These three quantities depend on age. The final curves of percentiles are produced by three smooth curves representing *L* (Lambda, skewness), *M* (Mu, median), and *S* (Sigma, coefficient of variation) (*LMS*) [7]. Descriptive statistics for each quarter year (e.g., 3–8 m, etc.) within sex were calculated by SPSS version 15.0 (Chicago, IL, USA).

## Results

There were 1,471 (50 %) boy and 1,476 (50 %) girl infants. The median waist circumference values increased with age for both study groups.

The descriptive statistics for waist circumference for boys and girls by age are given in Table 1. In Tables 2 and 3, the selected waist circumference percentiles including 3rd, 10th 25th, 50th, 75th, 90th, and 97th percentiles are shown for each age and gender. The *LMS* parameters for waist circumference are shown in Table 4. The mean and 1, 2, 3 standard deviations added and subtracted WC for each age group and gender are shown in Tables 5 and 6.

**Table 1:** Descriptive statistics for waist circumference for boys and girls by age

Age	Boys		Girls	
	*n*	Median (min–max)	*n*	Median (min–max)
0–28 days	108	31.00 (28.00–34.00)	108	31.00 (26.00–40.00)
28 days–3 months	49	38.00 (30.50–44.80)	44	37.50 (31.50–45.00)
3–<6 m	70	41.00 (33.00–48.00)	63	40.00 (34.50–49.50)
6–<9 m	78	42.00 (35.05–53.05)	64	41.13 (32.00–49.00)
9–<12 m	71	44.00 (35.10–52.00)	75	42.50 (34.60–51.25)
12–<15 m	58	43.63 (39.50–51.00)	56	43.50 (38.50–50.00)
15–<18 m	57	44.00 (40.30–50.50)	61	44.00 (39.00–50.00)
18–<21 m	54	44.00 (39.50–51.00)	73	43.80 (39.00–50.00)
21–<24 m	57	45.00 (39.50–51.00)	66	45.00 (38.10–50.00)
24–<27 m	36	46.40 (41.00–52.00)	52	46.00 (41.00–53.00)
27–<30 m	56	48.00 (41.50–54.00)	48	47.00 (41.00–52.00)
30–<33 m	57	48.00 (41.00–54.00)	48	46.70 (41.00–51.00)
33–<36 m	56	48.00 (41.00–54.00)	44	49.00 (43.00–53.30)
36–<39 m	50	48.75 (45.00–55.50)	51	49.00 (43.00–57.00)
39–<42 m	37	50.00 (45.00–56.75)	53	48.50 (43.00–57.00)
42–45 m	41	50.00 (45.00–55.20)	40	48.00 (42.50–58.00)
45–<48 m	51	49.50 (45.50–56.40)	44	50.75 (42.50–58.00)
48–<51 m	38	50.90 (45.00–57.00)	44	50.00 (45.00–58.00)
51–<54 m	41	51.00 (45.00–62.00)	45	51.00 (45.00–61.30)
54–<57 m	46	51.70 (46.00–60.60)	60	52.00 (45.00–59.00)
57–<60 m	44	52.00 (48.00–61.00)	38	51.50 (45.80–61.50)
60–<63 m	54	52.75 (46.00–62.00)	22	50.65 (45.00–61.40)
63–<66 m	47	52.00 (46.00–63.00)	46	52.75 (46.00–61.00)
66–<69 m	46	52.00 (47.00–68.00)	57	52.00 (45.0–62.00)
69–<72 m	47	52.00 (46.00–66.00)	55	52.00 (45.00–61.00)
72–<75 m	42	54.00 (48.00–64.00)	41	51.60 (47.00–64.60)
75–<78 m	30	51.75 (47.00–60.00)	35	51.50 (47.00–62.00)
78–<81 m	30	53.50 (47.00–66.00)	24	53.00 (48.00–66.00)
81–<84 m	20	55.00 (47.10–66.00)	19	53.00 (47.00–66.00)

**Table 2:** Smoothed age-specific waist circumference percentile values for boys

Age	3p	5p	10p	25p	50p	75p	90p	95p	97p
0–28 days	26.6	27.3	28.2	29.7	31.2	32.5	33.6	34.3	34.7
28 days–3 months	32.2	32.8	33.8	35.4	37.2	39.0	40.5	41.4	42.0
3–<6	34.9	35.5	36.5	38.2	40.0	42.0	43.7	44.8	45.5
6–<9	36.5	37.1	38.1	39.8	41.7	43.7	45.5	46.7	47.4
9–<12	37.7	38.3	39.2	40.9	42.8	44.9	46.8	48.0	48.7
12–<15	38.6	39.2	40.2	41.8	43.7	45.8	47.7	48.9	49.7
15–<18	39.4	40.0	40.9	42.6	44.5	46.6	48.5	49.8	50.6
18–<21	40.1	40.7	41.6	43.3	45.2	47.3	49.3	50.5	51.4
21–<24	40.8	41.4	42.3	43.9	45.9	48.0	50.0	51.3	52.2
24–<27	41.4	42.0	42.9	44.6	46.5	48.6	50.7	52.0	52.9
27–<30	42.0	42.6	43.5	45.2	47.1	49.3	51.4	52.7	53.6
30–<33	42.6	43.2	44.1	45.7	47.7	49.9	52.0	53.4	54.4
33–<36	43.1	43.7	44.6	46.2	48.2	50.4	52.6	54.1	55.1
36–<39	43.6	44.2	45.1	46.7	48.7	51.0	53.2	54.7	55.7
39–<42	44.1	44.6	45.6	47.2	49.2	51.5	53.8	55.3	56.4
42–45	44.5	45.1	46.0	47.6	49.7	52.0	54.4	55.9	57.0
45–<48	44.9	45.5	46.4	48.0	50.1	52.5	54.9	56.5	57.6
48–<51	45.3	45.8	46.8	48.4	50.5	52.9	55.4	57.1	58.2
51–<54	45.6	46.2	47.1	48.8	50.9	53.3	55.9	57.6	58.8
54–<57	46.0	46.5	47.5	49.1	51.3	53.7	56.3	58.1	59.3
57–<60	46.3	46.9	47.8	49.5	51.6	54.1	56.8	58.6	59.9
60–<63	46.6	47.2	48.1	49.8	51.9	54.5	57.2	59.1	60.4
63–<66	46.9	47.4	48.4	50.1	52.3	54.8	57.6	59.5	60.9
66–<69	47.2	47.7	48.6	50.4	52.5	55.2	58.0	60.0	61.4
69–<72	47.4	48.0	48.9	50.6	52.8	55.5	58.4	60.4	61.9
72–75	47.7	48.2	49.2	50.9	53.1	55.8	58.7	60.8	62.3
75–78	47.9	48.5	49.4	51.1	53.4	56.1	59.1	61.2	62.8
78–81	48.1	48.7	49.6	51.4	53.6	56.4	59.4	61.6	63.2
81–84	48.4	48.9	49.9	51.6	53.9	56.6	59.7	62.0	63.7

**Table 3:** Smoothed age-specific waist circumference percentile values for girls

Age	3p	5p	10p	25p	50p	75p	90p	95p	97p
0–28 days	27.0	27.6	28.4	29.8	31.4	33.2	34.9	36.0	36.7
28 days–3 months	32.0	32.6	33.5	35.0	36.8	38.7	40.6	41.7	42.5
3–<6	34.5	35.1	36.0	37.6	39.5	41.5	43.3	44.5	45.2
6–<9	36.1	36.7	37.6	39.3	41.1	43.1	45.0	46.1	46.9
9–<12	37.3	37.9	38.8	40.4	42.3	44.3	46.2	47.3	48.1
12–<15	38.2	38.8	39.7	41.4	43.3	45.3	47.2	48.3	49.1
15–<18	39.0	39.6	40.5	42.2	44.1	46.1	48.0	49.2	50.0
18–<21	39.7	40.3	41.2	42.9	44.8	46.9	48.8	50.0	50.8
21–<24	40.3	40.9	41.9	43.5	45.5	47.6	49.6	50.8	51.6
24–<27	40.9	41.5	42.5	44.2	46.1	48.3	50.3	51.5	52.4
27–<30	41.5	42.1	43.1	44.7	46.7	48.9	51.0	52.3	53.2
30–<33	42.0	42.6	43.6	45.3	47.3	49.5	51.6	53.0	53.9
33–<36	42.5	43.1	44.1	45.8	47.8	50.1	52.2	53.6	54.6
36–<39	42.9	43.5	44.5	46.2	48.3	50.6	52.8	54.3	55.2
39–<42	43.3	43.9	44.9	46.7	48.8	51.1	53.4	54.9	55.9
42–45	43.7	44.3	45.3	47.1	49.2	51.6	53.9	55.5	56.5
45–<48	44.1	44.7	45.7	47.4	49.6	52.0	54.4	56.0	57.1
48–<51	44.4	45.0	46.0	47.8	50.0	52.4	54.9	56.5	57.6
51–<54	44.7	45.3	46.3	48.1	50.3	52.8	55.4	57.0	58.2
54–<57	45.0	45.6	46.6	48.4	50.7	53.2	55.8	57.5	58.7
57–<60	45.3	45.9	46.9	48.7	51.0	53.6	56.2	58.0	59.2
60–<63	45.5	46.2	47.2	49.0	51.3	53.9	56.6	58.4	59.7
63–<66	45.8	46.4	47.4	49.3	51.6	54.2	57.0	58.8	60.1
66–<69	46.0	46.7	47.7	49.5	51.9	54.5	57.3	59.2	60.6
69–<72	46.3	46.9	47.9	49.8	52.1	54.8	57.7	59.6	61.0
72–75	46.5	47.1	48.1	50.0	52.4	55.1	58.0	60.0	61.4
75–78	46.7	47.3	48.3	50.2	52.6	55.4	58.4	60.4	61.8
78–81	46.9	47.5	48.6	50.4	52.8	55.7	58.7	60.8	62.2
81–84	47.1	47.7	48.8	50.6	53.1	55.9	59.0	61.1	62.6

**Table 4:** The power of a Box–Cox transformation (L), the median (M), and the coefficient of variation (S) for waist circumference for both genders

Age	Boys			Girls		
	L	M	S	L	M	S
0–28 days	3.030	31.185	0.067	−0.192	31.449	0.081
28 days–3 months	1.360	37.206	0.070	−0.147	36.813	0.075
3–<6	0.611	40.042	0.070	−0.098	39.488	0.072
6–<9	0.201	41.691	0.070	−0.078	41.134	0.069
9–<12	−0.061	42.835	0.068	−0.099	42.320	0.068
12–<15	−0.258	43.731	0.067	−0.143	43.266	0.067
15–<18	−0.424	44.503	0.066	−0.207	44.079	0.066
18–<21	−0.578	45.210	0.066	−0.288	44.814	0.066
21–<24	−0.729	45.879	0.065	−0.383	45.498	0.066
24–<27	−0.881	46.517	0.065	−0.489	46.141	0.066
27–<30	−1.034	47.123	0.065	−0.600	46.742	0.066
30–<33	−1.188	47.695	0.064	−0.715	47.306	0.066
33–<36	−1.342	48.236	0.064	−0.833	47.833	0.066
36–<39	−1.493	48.747	0.064	−0.949	48.324	0.067
39–<42	−1.642	49.229	0.065	−1.065	48.783	0.067
42–45	−1.787	49.684	0.065	−1.178	49.212	0.068
45–<48	−1.927	50.114	0.065	−1.289	49.614	0.068
48–<51	−2.062	50.519	0.065	−1.396	49.991	0.069
51–<54	−2.192	50.903	0.066	−1.500	50.346	0.069
54–<57	−2.318	51.266	0.066	−1.600	50.680	0.070
57–<60	−2.438	51.611	0.066	−1.697	50.996	0.070
60–<63	−2.553	51.938	0.067	−1.790	51.295	0.070
63–<66	−2.664	52.250	0.067	−1.880	51.580	0.071
66–<69	−2.770	52.548	0.067	−1.967	51.852	0.071
69–<72	−2.871	52.833	0.067	−2.051	52.112	0.072
72–75	−2.968	53.107	0.068	−2.131	52.362	0.072
75–78	−3.062	53.371	0.068	−2.209	52.602	0.072
78–81	−3.152	53.625	0.068	−2.284	52.834	0.073
81–84	−3.239	53.870	0.069	−2.356	53.057	0.073

**Table 5:** The mean and 1, 2, and 3 standard deviations added and subtracted WC for boys

Age	−3 SD	−2 SD	−1 SD	mean	+1 SD	+2 SD	+3 SD
0–28 days	36.6	27.1	29.0	30.9	32.8	34.7	36.6
28 days–3 months	47.5	31.5	34.7	37.9	41.1	44.3	47.5
3–<6 m	49.8	34.8	37.8	40.8	43.8	46.8	49.8
6–<9 m	51.4	35.4	38.6	41.8	45.0	48.2	51.4
9–<12 m	53.7	36.7	40.1	43.5	46.9	50.3	53.7
12–<15 m	52.6	38.1	41.0	43.9	46.8	49.7	52.6
15–<18 m	52.5	39.0	41.7	44.4	47.1	49.8	52.5
18–<21 m	53.0	38.5	41.4	44.3	47.2	50.1	53.0
21–<24 m	52.4	39.4	42.0	44.6	47.2	49.8	52.4
24–<27 m	55.4	40.9	43.8	46.7	49.6	52.5	55.4
27–<30 m	55.5	42.5	45.1	47.7	50.3	52.9	55.5
30–<33 m	55.9	41.9	44.7	47.5	50.3	53.1	55.9
33–<36 m	56.6	42.1	45.0	47.9	50.8	53.7	56.6
36–<39 m	56.9	43.9	46.5	49.1	51.7	54.3	56.9
39–<42 m	59.9	43.4	46.7	50.0	53.3	56.6	59.9
42–45 m	58.0	44.0	46.8	49.6	52.4	55.2	58.0
45–<48 m	58.0	45.0	47.6	50.2	52.8	55.4	58.0
48–<51 m	61.1	44.6	47.9	51.2	54.5	57.8	61.1
51–<54 m	62.1	44.1	47.7	51.3	54.9	58.5	62.1
54–<57 m	64.1	44.6	48.5	52.4	56.3	60.2	64.1
57–<60 m	61.6	46.6	49.6	52.6	55.6	58.6	61.6
60–<63 m	64.4	45.4	49.2	53.0	56.8	60.6	64.4
63–<66 m	64.1	45.1	48.9	52.7	56.5	60.3	64.1
66–<69 m	70.8	43.3	48.8	54.3	59.8	65.3	70.8
69–<72 m	64.8	44.3	48.4	52.5	56.6	60.7	64.8
72–<75 m	65.4	46.4	50.2	54.0	57.8	61.6	65.4
75–<78 m	63.6	45.1	48.8	52.5	56.2	59.9	63.6
78–<81 m	68.7	45.7	50.3	54.9	59.5	64.1	68.7
81–<84 m	69.6	45.6	50.4	55.2	60.0	64.8	69.6

**Table 6:** The mean and 1, 2, and 3 standard deviations added and subtracted WC for girls

Age	−3 SD	−2 SD	−1 SD	Mean	+1 SD	+2 SD	+3 SD
0–28 days	23.5	26.1	28.7	31.3	33.9	36.5	44.3
28 days–3 months	28.3	31.4	34.5	37.6	40.7	43.8	53.1
3–<6 m	31.5	34.4	37.3	40.2	43.1	46.0	54.7
6–<9 m	31.9	35.0	38.1	41.2	44.3	47.4	56.7
9–<12 m	33.4	36.5	39.6	42.7	45.8	48.9	58.2
12–<15 m	34.9	37.8	40.7	43.6	46.5	49.4	58.1
15–<18 m	35.8	38.6	41.4	44.2	47.0	49.8	58.2
18–<21 m	36.5	39.0	41.5	44.0	46.5	49.0	56.5
21–<24 m	36.8	39.5	42.2	44.9	47.6	50.3	58.4
24–<27 m	37.4	40.4	43.4	46.4	49.4	52.4	61.4
27–<30 m	37.7	40.7	43.7	46.7	49.7	52.7	61.7
30–<33 m	39.1	41.6	44.1	46.6	49.1	51.6	59.1
33–<36 m	37.7	41.3	44.9	48.5	52.1	55.7	66.5
36–<39 m	40.7	43.5	46.3	49.1	51.9	54.7	63.1
39–<42 m	39.0	42.4	45.8	49.2	52.6	56.0	66.2
42–45 m	38.5	41.9	45.3	48.7	52.1	55.5	65.7
45–<48 m	38.8	42.7	46.6	50.5	54.4	58.3	70.0
48–<51 m	41.2	44.3	47.4	50.5	53.6	56.7	66.0
51–<54 m	39.9	43.7	47.5	51.3	55.1	58.9	70.3
54–<57 m	40.5	44.2	47.9	51.6	55.3	59.0	70.1
57–<60 m	39.0	43.4	47.8	52.2	56.6	61.0	74.2
60–<63 m	40.3	44.0	47.7	51.4	55.1	58.8	69.9
63–<66 m	42.0	45.6	49.2	52.8	56.4	60.0	70.8
66–<69 m	38.8	43.2	47.6	52.0	56.4	60.8	74.0
69–<72 m	39.5	43.7	47.9	52.1	56.3	60.5	73.1
72–<75 m	38.5	43.1	47.7	52.3	56.9	61.5	75.3
75–<78 m	42.3	45.7	49.1	52.5	55.9	59.3	69.5
78–<81 m	41.1	45.5	49.9	54.3	58.7	63.1	76.3
81–<84 m	39.7	44.2	48.7	53.2	57.7	62.2	75.7

**Fig. 1:**
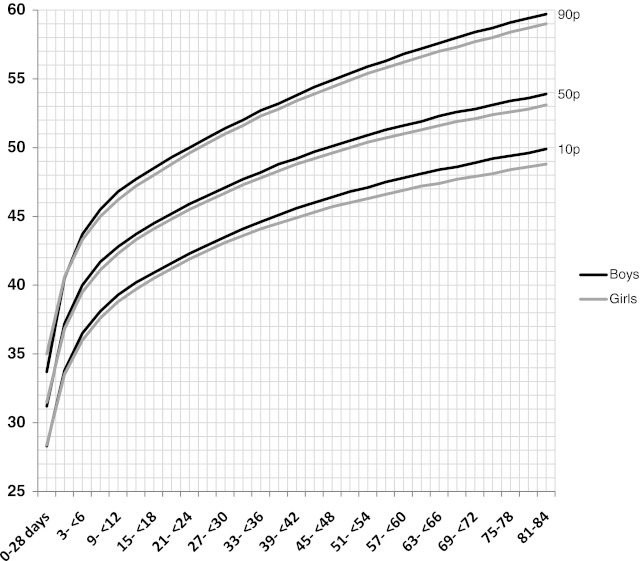
The comparison of 10th, 50th, and 90th percentile between genders

In comparison to 10th, 50th, and 90th percentiles between genders, boys’ WC were longer than girls’ after the infancy period (Fig. 1).

**Fig. 2:**
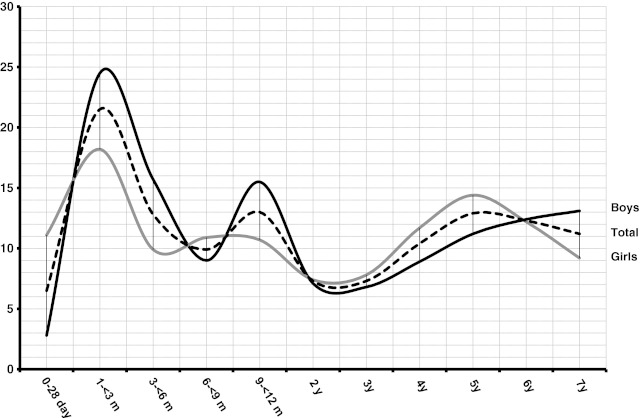
The frequency of WC higher than 90th percentile between each gender

**Fig. 3:**
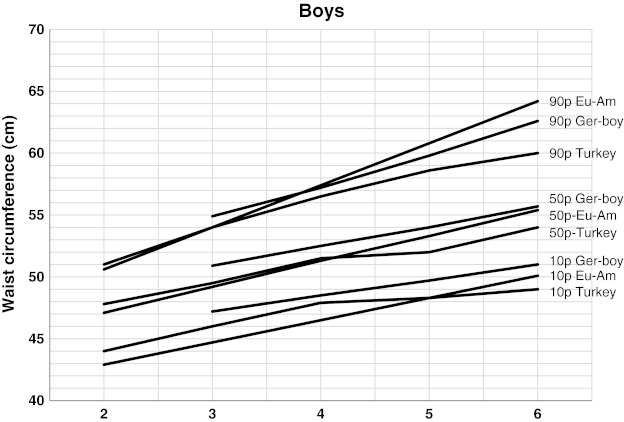
The comparison of 10th, 50th, and 90th percentiles of our WC (Turkey) with the preschool children of German (*Ger*) and European American (*Eu Am*) for boys

**Fig. 4:**
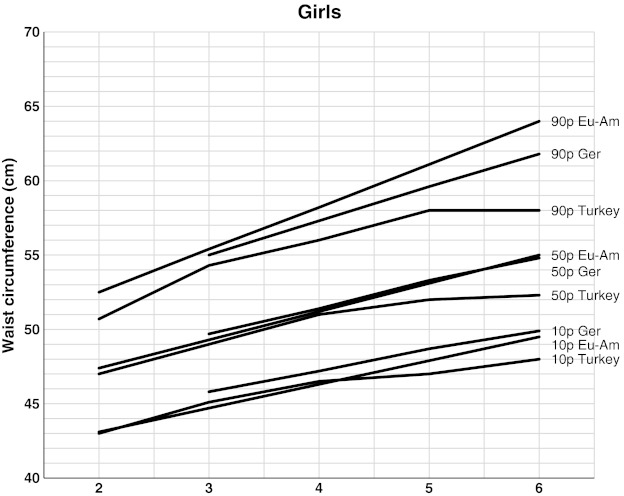
The comparison of 10th, 50th, and 90th percentiles of our WC (Turkey) with the preschool children of German (*Ger*) and European American (*Eu Am*) for girls

We also compared the frequency of WC higher than 90th percentile between each gender to reveal the fluctuations in abdominal obesity prevalence (Fig. 2). The 10th, 50th, and 90th percentiles of our study were compared with other studies, the methodologies of which are similar to ours (Figs. 3 and 4).

## Discussion

This is the first cross-sectional study for age- and gender-specific references of 0- to 6-year-old Turkish children. In addition to finding a slightly low WC in girls compared with boys, in comparison with other studies in similar age groups, the WC of boys and girls were lower. The age- and gender-specific prevalence of abdominal obesity was also calculated. Because of difficulty in sampling in infancy and preschool children and the high number of studies about obesity prevalence in school children and adolescence, we concentrated on infants and preschool children [22, 42].

Early-onset obesity (<6 years) not only increases the risk of metabolic and cardiovascular disorders in adulthood but also indicates that the risk of adult obesity would be increased by at least 25–50 % [14, 25, 31]. Then, this study would at least provide the opportunity of determining the validity of the preceding statement by comparing with future similar cross-sectional studies in our population.

There is a moderate correlation between obesity in early childhood and in adulthood [16]. In a report by Nader et al., the authors stated that preschool children whose BMIs were higher than 50p would have six-times-higher risk of overweight in later childhood [28]. In addition to these findings, obesity at the age of 5 was proposed as the predictor of metabolic status at 9 years old [42]. Then, early interventions to treat obesity would not only have a short time but would also have a long period of consecutions [2]. WC references which were determined in this study would be useful to determine metabolic risks in early childhood since a similar study was not done in our population.

It is known that there is a decrease in BMI from infancy to 6 years old, the so-called adiposity rebounds, but because of individual variations during this period any two children who have the same BMI may be at different levels of adiposity. Since the onset of adiposity rebound varies individually in this period, children whose adiposity onset is early may have similar BMIs with the relatively late-onset one [33]. Thus, in preschool children (<6 years old), we may conclude that the use of BMI to predict adiposity is not reliable. Additionally, in this period, non-uniform segmental growth leads the need to use WC but not BMI since BMI reflects total body fat content but not body fat distribution [3, 4, 32]. BMI may define large central fat deposits as normal, while it has poor sensitivity to define central obesity [32]. Therefore, WC prevents misclassification for central fat deposit and minimizes individual variation [39]. Since WC in children has a good correlation with insulin resistance, it is considered as a useful tool to predict the risk of developing metabolic and cardiovascular complications [23]. The correlation between WC and central adiposity is also confirmed by dual-energy X-ray absorptiometry [39]. The rationale of this study depends on this hypothesis that segmental fat distribution is best reflected by WC compared with BMI.

The primary contribution of this study is to provide the first WC references in Turkish preschool children to our knowledge. Our findings indicate that the increase in WC through early childhood is faster than 1.5 to 6 years. According to our findings, predominance in central adiposity for boys compared with girls may be an indicator of sexual dimorphism in early childhood in accordance with previous studies [15, 38].

Although there are different cutoffs to define central obesity, we used WC > 90th percentile since it was proposed as the prerequisite in the definition of metabolic syndrome. In addition to smoothed references (3rd, 10th 25th, 50th, 75th, 90th, and 97th percentiles), we presented all descriptive characteristics of our data (mean, median, standard deviations) to let other researchers make comments on our results. The cited methods to define cutoff for WC can be stated as: *z* score ≥1.3 by Fredrick, *z* score ≥1.5 by Taylor, at >75th by Moreno, and age- and sex-specific 90th by Katzmarzyk [12, 18, 26, 39].

Increased WC > 90th percentile is proposed by NCEP-ATP III and IDF, but cutoff for WC to define metabolic syndrome is lacking before 10 years old [9, 40, 41]. However, in recent studies, to diagnose metabolic risk, it is suggested that modified IDF and NCEP criteria can be used in the pre-pubertal period (6–10 years) [6, 14, 45]. In these modified criteria, WC >90th percentile was accepted as cutoff value to define metabolic risk at older than 10 years. Although cutoff for abdominal obesity is not determined under 6 years, WC >90th percentile may be speculated at this level of knowledge [6, 14]. According to this criterion, we found total abdominal obesity prevalence as 10.4 % (boys 10.1 %, girls 10.7 %), where age-adjusted prevalence values were also given.

We also compared the frequency of WC higher than 90th percentile between each gender and found significant fluctuations until the age of 2 years. After 2 years, WC showed a small decline until 3 years and it was followed by a gradual increase until 6 years in both genders, where abdominal obesity was higher in girls during this period (Fig. 2). The observation that the increase in abdominal obesity was shifted towards the early years can be interpreted as early rebound. Although BMI and WC do not completely reflect the same metabolic parameters, early onset of abdominal obesity may require the onset of rebound obesity for BMI. Since WC reflects primarily the body fat distribution but not total body fat, inconsistent obesity rebounds in WC and BMI must be evaluated separately.

There are few studies about WC percentiles for children under 6 years old. In a study conducted in Sweden on children less than 5 year old, SDS values are calculated for WC, but not percentiles. This prevented us to make a comparison of percentiles [34]. In another study conducted in 3–16-year-old Indian children, there were calculated percentiles which can be compared with our references. We could not make a comparison with those since they were not Caucasian [21]. We selected two previous studies providing WC references with a similar method in the same age of Caucasians. One of them was calculated WC percentiles in 3–11-year-old German children. The other one was a study conducted in 2–18-year-old European American children in USA [11, 37]. In comparing our data with these two studies, we found that our WC references were significantly lower than those (Figs. 3 and 4). These differences may be explained by dissimilarities in ethnic, geographical, and nutritional behaviors as well as lifestyle. Even in the pre-pubertal period, these dissimilarities may be observed [11].

We consider that behaviors related to sedentary activities such as watching television and/or playing with electronic games are established in the preschool period. Our data can then be used to determine the risk of central obesity in early childhood. We can conclude that WC together with BMI is a useful clinical tool to detect preschool-age children who may be at a higher risk for cardiovascular diseases.
